# Association between adequacy of antenatal care and neonatal outcomes in Rwanda: a cross-sectional study design using the Rwanda demographic and health surveys

**DOI:** 10.1186/s12913-023-10345-6

**Published:** 2023-12-08

**Authors:** Gérard Uwimana, Mohamed Elhoumed, Mitslal Abrha Gebremedhin, Qi Qi, Mougni Mohamed Azalati, Liang Wang, Lingxia Zeng

**Affiliations:** 1 Department of Epidemiology and Biostatistics, School of Public Health, Xi’an Jiaotong University Health Science Center, No. 76, Yanta West Road, Xi’an, Shaanxi Province 710061 People’s Republic of China; 2 National Institute of Public Health Research (INRSP), Nouakchott BP. 695, Nouakchott, Mauritania; 3 Center for Chronic Disease Control and Prevention, Global Health Institute, Xi’an Jiaotong University, Xi’an, China; 4 Key Laboratory for Disease Prevention and Control and Health Promotion of Shaanxi Province, Xi’an, China

**Keywords:** Antenatal care, Neonatal outcomes, Child health, Rwanda

## Abstract

**Background:**

Maternal and neonatal health services are life-saving interventions for neonatal health outcomes. As Rwanda endeavors to accomplish sustainable development goals, adequate ANC is essential to lessen of neonatal mortality. The utilization of ANC continues to be inadequate and high neonatal mortality rate persevere in Rwanda. Understanding the direct and indirect factors that affect newborn health outcomes is necessary for well-targeted interventions. However, few studies had been conducted in Rwanda to evaluate the importance of ANC in improving neonatal health. This study therefore assessed the association between ANC and neonatal outcomes.

**Methods:**

The Demographic and Health Surveys (DHS) are household surveys that are cross-sectional, nationally representative, and used to collect data on population, health, and nutrition. Data from the 2010,2015 and 2020 Rwanda Demographic and Health Surveys (RDHS) were used. The study involved 17,747 women between the ages of 15 and 49 who had a single live birth and at least one ANC visit in five years prior to each survey. Bivariate and multivariable logistic regression, a survey adjusted for clusters at multiple level, and the estimation of adjusted odds ratios (aOR) and 95% confidence intervals were used to evaluate the relationship between the outcome and independent variables.

**Results:**

Out of 17,747 women ;7638(42.91%) of the mothers had adequate ANC visits and low birth weight (LBW) was found among 833(4.63%) neonates. The birth of a LBW baby (aOR:4.64;95%CI:3.19,6.74) was directly related to increased odds of neonatal death. Mothers aged 20–34 years (aOR:0.40; 95%CI:0.20,0.81), a preceding birth interval of 24months or greater (aOR:0.41:95%CI:0.28,0.60), baby being female (aOR:0.72; 95%CI:0.54,0.96), having adequate ANC visits (aOR:0.64;95% CI:0.46,0.89) and the birth order of the newborn being ranked second or third (aOR:0.60; 95%CI:0.38,0.95) were negatively associated with neonatal death.

**Conclusion:**

Health education programs targeting teen and primigravida mothers should be encouraged. Among the newborn survival interventions, addressing short birth intervals and the effective management of LBW cases should be explored. The findings confirm the fundamental importance of adequate ANC in the neonatal survival.

**Supplementary Information:**

The online version contains supplementary material available at 10.1186/s12913-023-10345-6.

## Introduction

Antenatal care (ANC) refers to the treatment given to pregnant women and adolescent girls by skilled health-care professionals in order to maximise the health of both mother and child throughout pregnancy [1]. Neonatal death, defined as death within the first 28 days of life, is one of the most pressing public health issues in low and middle-income countries (LMICs) [2–4]. In 2020, an estimated 2.4 million neonatal deaths were reported, reflecting a global burden of 17 neonatal deaths per 1000 live births, and the majority of these neonatal deaths took place in LMICs [2–4]. More than three-quarters (80%) of all newborn mortality occur in Central, South Asia, and Sub-Saharan Africa (SSA) [5]. With one in every 38 neonates dying before the age of one month, SSA has the highest rate of neonatal mortality [5].

Rwanda’s neonatal mortality rate is estimated to be 18 per 1000 live births, far higher than the UN Sustainable Development Goal (SDG) 3.2, which is to reduce neonatal mortality to less than 12 per 1000 live births [5–7].

The utilization of maternal health care services in Rwanda is below average except for the health facility delivery which is nearly 90% [8]. The adequacy of ANC and the utilization of postnatal care services within seven days following delivery is 47.2% and 12.8% respectively [8]. Nonetheless, these services are essential for identifying the fetus and maternal risk indications during pregnancy, labor, and postpartum [9, 10]. According to a study done in 57 LMICs, both adequate ANC visits and timing of first ANC were linked to lower odds of neonatal mortality [11]. A study done in Kenya, a country in the region revealed that missing any ANC was linked to neonatal mortality [12]. The 2016 World Health Organization (WHO) guidelines on ANC recommend at least 8 contacts for every pregnant woman; however, Rwanda still implements the 2001 policy which only recommended 4 visits [8]. The utilization of these services in Rwanda is challenged by individual, socioeconomic and community level factors [13–15]. Women’s unenlightenment on pregnancy complications [16] and the perceived barriers to utilizing health care [15] were found to be hindering ANC attendance in Rwanda.

Other challenges to the use of the maternal healthcare services include unskilled and poor attitude of health providers [17, 18]. A recent study found that the COVID-19 in Rwanda dramatically reduced the use of maternal and child health services [19].

The determinants of neonatal outcomes and maternal health-care utilization are always based on methods that assume direct rather than indirect relationships [20, 21]. Given that ANC is linked to better maternal and foetal health indicators and that Rwanda continues to have a high neonatal mortality rate, our study’s exposure is ANC and its outcome is neonatal death (death within 28 days of birth). We also studied other health services factors such as iron supplementation and tetanus injections during pregnancy to examine their association with neonatal outcomes in Rwanda settings. Few studies had been conducted at Rwanda country level to examine the associations between adequate ANC on neonatal death. We sought to breach this gap by exploring the associations between adequacy of ANC, various confounders and neonatal outcomes. This study adds on the scant evidence already available to help devise retention strategies for ANC attendance in the continuum of care and assess ANC services and their links to neonatal survival.

## Methods

### Study settings

Rwanda is landlocked country in the central eastern Africa. Its landscape is characterized by hills and with its 503 persons per kilometer square is ranked in the top 3 most densely populated countries in Africa [22]. The health system in Rwanda is organized as a three-level pyramid consisting of central, intermediate, and peripheral levels [23]. The central level includes the directorates of the Ministry of Health and the national reference hospitals. The intermediate level is represented by health districts and district hospitals. The peripheral level is represented by health centers and health posts, which include community health workers [23].

The central level, based in the capital city of Kigali, is responsible for the development of health policy, strategy, monitoring &evaluation as well as to coordinate resources at the national level. The intermediate, or district, level is represented by health district administration and the referral district hospitals. The district hospital is responsible for managing all health problems for a well-defined population, supervises health centres and communities and reports to the health district administration. The peripheral level located at the sector and cell levels is the operational unit represented by the health centres and the community-based organizations. It is comprised of first-level health facilities, particularly health centres and health posts. With participation from the community, the health facility plans, coordinates, and carries out health activities in its catchment area [23].

Most of women receive ANC at health centres, with the clinic and referral hospital only being utilized for particular tests like ultrasounds [24]. Health centers are understaffed and rural women have difficulties accessing them due to poor road networks in a hilly landscape, this negatively impact the ANC attendance as well as referral systems [25]. Inadequate ANC visits, poor attitude of health workers and a weak referral system in place might be contributing to a high neonatal mortality.

### Study design and data source

This study is a cross-sectional study design that used secondary data from three waves of Rwanda Demographic and Health Survey (RDHS). The three waves are the RDHS 2010, RDHS 2015 and RDHS 2020.The RDHS is a cross-sectional survey that employs a two-stage sampling design to get a nationally representative household sample. In the first step, clusters (villages) are chosen from a list of all clusters in the country. Selecting households within each cluster is done in the second stage. Over the three waves, the women’s response has been high, exceeding 99%. The RDHS gathers information on mother and child health services during a time frame within the five years prior to the survey. More specifics to sampling design, sample size, study instruments, data collection, how informed consent was obtained, and other related procedure are described elsewhere [8]. The RDHS data are available at Measure DHS website at https://dhsprogram.com/data/available-datasets.cfm.

### Analytic sample

**Fig. 1:**
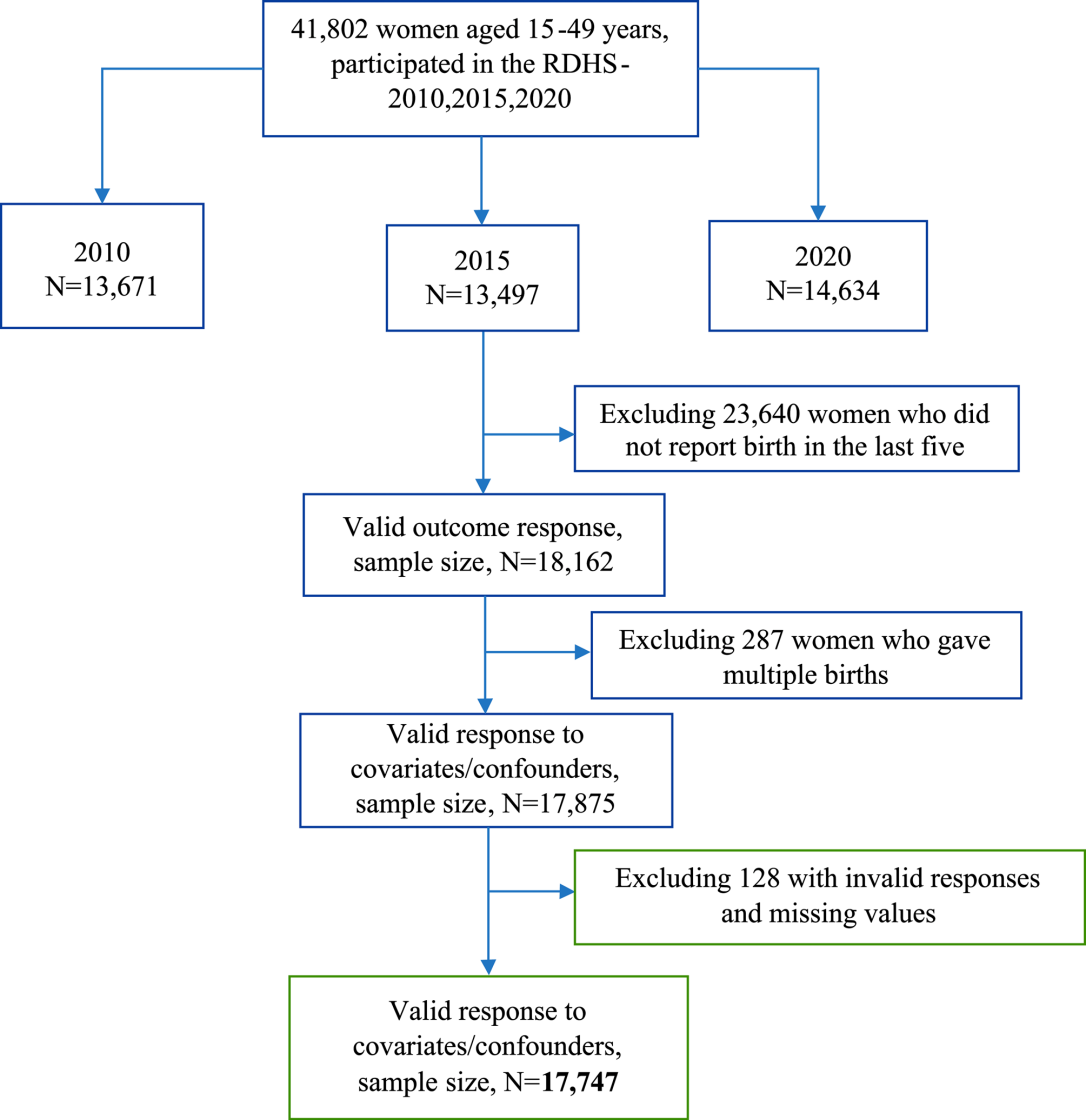
Flow chart of the analytic sample selection

The 2010, 2015, and 2020 RDHS birth recode (BR) datasets were merged for the purposes of this study using accepted standards for handling DHS data. Women aged 15 to 49 years’ old who had a single live birth in the five years prior to each survey and had at least one ANC visit answered questions about ANC were included in this sample. Women with missing values or invalid responses to the main exposure, outcome, and possible confounders, such as “don’t know”, were drawn out. 17,747 of the 41,802 women who participated in the survey met the criteria for inclusion. More information is shown in Fig. 1.

### Study variables

#### Outcome and exposure

The study had one dependent variable which is neonatal death, which was defined as the death of a newborn 28 days following birth. Neonatal death was dichotomous and coded 0 and 1 for no and yes respectively.

The main exposure variable was adequacy of ANC. Four ANC visits are considered adequate (coded 1) according to the policy in use in Rwanda during the time of the study. The visits were considered inadequate when they were less than four (coded 0).

We considered as control variables several socioeconomic, demographic and health service factors for their relevance in the uptake of ANC and the effect on neonatal death. These factors were adapted from Andersen’s behavioral model, Mosley and Chen [26]. Many studies have made use of the Andersen’s behavioral model and the analytical framework by Mosley and Chen to study the determinants of the maternal health services utilization and birth outcomes [26–28].

Categories were created for numerical values like age and birth order. Maternal age in years was tabulated into groups (15–19 years, 20–34 years, 35–49 years); the birth order of the baby was into three categories (1st, 2nd -3rd ,4th and above). Using principal component analysis, the household wealth index was created from data on the ownership of long-lasting assets, access to infrastructure and utilities, and dwelling features. Each woman was into five categories (poorest, poorer, middle, richer, and richest) depending on a household asset score, comprising 20% of the population. Later, these five categories were translated into three categories (poor, middle, and rich).

### Statistical analyses

Stata v17.0 [29] was used for all of the statistical analysis. Descriptive statistics for the sociodemographic characteristics of the study participants by survey year were generated using the frequency and percentage as indicated in Table 1. We used chi-square tests to establish demographic and socio-economic factors associated with the outcome variable. Potential factors with p < 0.20 were retained for multivariable logistic regression in the final model. To assess the goodness of fit of the model we used AIC (Akaike’s information criterion). When covariates were found to be collinear, utilising the variance inflation factor (VIF > 4), the variable most correlated with the outcome variable was retained. We carried out all analyses utilizing the survey module “svyset” stata commands, taking into account clustering, stratification, and sample weight.

**Table 1:** Baseline characteristics of respondents and outcome variables

	Year 2010	Year 2015	Year 2020	Combined
**Variables**	N(%)	N(%)	N(%)	N(%)
**Adequate ANC**				
No	3859(63.90)	3220(55.83)	3030(51.42)	10,109(57.09)
Yes	2181(36.10)	2568(44.17)	2889(48.58)	7638(42.91)
**Type of residence**				
Urban	875(12.71)	1289(17.02)	1267(17.73)	3431(15.80)
Rural	5165(87.29)	4499(82.98)	4652(82.27)	14,316(84.20)
**Water sources**				
Improved	4342(73.11)	4126(71.03)	2794(67.05)	11,262(70.77)
Unimproved	1548(26.89)	1575(28.97)	1346(32.95)	4469(29.23)
**Maternal education**				
No education	1096(18.51)	813(14.46)	626(10.75)	2535(14.59)
Primary	4313(71.71)	4117(71.94)	3839(64.38)	12,269(69.33)
Secondary & higher	631(9.78)	858(13.60)	1454(24.86)	2943(16.08)
**Married/partnered**				
No	1008(16.52)	1162(19.78)	1130(18.99)	3300(18.41)
Yes	5032(83.48)	4626(80.22)	4789(81.01)	14,447(81.59)
**Access to media**				
Not at all	485(8.11)	871(15.90)	1234(20.25)	2590(14.71)
Less than once a week	1529(25.51)	1483(25.68)	1108(18.32)	4120(23.15)
At least once a week	4026(66.39)	3426(58.42)	3577(61.43)	11,029(62.13)
**Wealth index**				
Poor	2617(44.01)	2550(44.86)	2533(41.71)	7700(43.51)
Middle	1172(19.66)	1087(19.76)	1130(19.53)	3389(19.65)
Rich	2251(36.33)	2151(35.38)	2256(38.76)	6658(36.83)
**Cooking fuel**				
Solid fuel	5275(88.47)	4908(85.45)	4629(79.65)	14,812(84.54)
Non-solid fuel	682(11.53)	783(14.55)	1196(20.35)	2661(15.46)
**Maternal age**				
15–19	127(2.17)	148(2.56)	122(2.05)	397(2.26)
20–34	4196(69.35)	4163(71.61)	3721(63.05)	12,080(67.97)
35–49	1717(28.48)	1477(25.84)	2076(34.90)	5270(29.77)
**Preceding birth interval**				
< 24months	866(14.32)	540(9.38)	596(10.30)	2002(11.36)
>=24months	5174(85.68)	5248(90.62)	5323(89.70)	15,745(88.64)
**Birth order**				
1st	1390(22.89)	1587(27.37)	1454(24.42)	4431(24.86)
2nd-3rd	2074(34.33)	2272(38.67)	2469(41.81)	6815(38.25)
4th&above	2576(42.78)	1929(33.96)	1996(33.76)	6501(36.88)
**Child wantedness**				
Wanted then	3454(57.38)	3498(60.66)	3481(58.50)	10,433(58.83)
Wanted later	1672(27.66)	1544(26.38)	1658(28.19)	4874(27.42)
Wanted no more	912(14.96)	745(12.95)	780(13.31)	2437(13.75)
**Sex of newborn**				
Male	3099(51.44)	2933(50.46)	3005(50.70)	9037(50.87)
Female	2941(48.56)	2855(49.54)	2914(49.30)	8710(49.13)
**Iron supplementation**				
No	1527(25.67)	1133(19.85)	1027(17.62)	3687(21.08)
Yes	4513(74.33)	4655(80.15)	4892(82.38)	14,060(78.92)
**Two tetanus injections**				
No	3899(65.07)	3805(66.17)	3861(65.44)	11,565(65.56)
Yes	2106(34.93)	1967(33.83)	2041(34.56)	6114(34.44)
**Low birthweight**				
No	5807(96.13)	5516(95.41)	5591(94.57)	16,914(95.37)
Yes	233(3.87)	272(4.59)	328(5.43)	833(4.63)

## Results

From the results in Table 1, it can be observed that adequate utilization of ANC increased over the three survey waves. Adequate utilization of ANC increased from 36.10% in 2010 through 44.17% in 2015 to 48.58% in 2020. Majority of the respondents were young women of 20–34 years (67.97%) and they were ranked poor (43.51%). Most of the interviewed women (84.20%) resided in rural areas and had a primary school education (69.33%). Almost a half of the newborns were males (50.87%).

Table 2 shows the distribution of adequacy of ANC by background characteristics and the results of the bivariable analysis for the relationship between the factors and neonatal death are shown in the rightest column. In bivariable analysis, variables significantly associated with neonatal death are maternal age, the preceding birth interval, birth order, and low birthweight.

**Table 2:** Adequacy of ANC by background characteristics and association with neonatal outcome

	Year 2010	Year 2015	Year 2020	Combined	P-Value
**Variables**	N(%)	N(%)	N(%)	N(%)	
**Type of residence**					
Urban	318(5.19)	448(7.62)	544(9.02)	1310(7.27)	
Rural	1889(30.91)	2150(36.55)	2388(39.56)	6428(35.65)	0.664
**Water sources**					
Improved	1591(26.68)	1824(31.48)	1360(32.81)	4775(30.03)	
Unimproved	549(9.21)	729(12.58)	652(15.74)	1930(12.14)	0.246
**Maternal education**					
No education	379(6.21)	320(5.44)	286(4.74)	985(5.46)	
Primary	1563(25.58)	1875(31.87)	1813(30.04)	5252(29.12)	
Secondary & higher	264(4.32)	403(6.86)	833(13.8)	1501(8.32)	0.160
**Married/partnered**					
No	319(5.22)	440(7.47)	460(7.62)	1219(6.76)	
Yes	1888(30.88)	2159(36.7)	2473(40.96)	6519(36.15)	0.209
**Access to media**					
Not at all	163(2.66)	391(6.65)	512(8.49)	1066(5.91)	
Less than once a week	540(8.84)	634(10.79)	500(8.27)	1674(9.29)	
At least once a week	1504(24.6)	1570(26.73)	1920(31.81)	4994(27.71)	0.605
**Wealth index**					
Poor	949(15.52)	1121(19.06)	1076(17.82)	3146(17.44)	
Middle	396(6.48)	536(9.11)	575(9.52)	1507(8.36)	
Rich	862(14.1)	941(16)	1282(21.24)	3085(17.11)	0.977
**Cooking fuel**					
Solid fuel	1934(32.1)	2219(38.35)	2286(38.53)	6439(36.29)	
Non-solid fuel	227(3.76)	328(5.68)	595(10.02)	1150(6.48)	0.350
**Maternal age**					
15–19	43(0.71)	62(1.06)	51(0.85)	157(0.87)	
20–34	1561(25.54)	1902(32.33)	1870(30.98)	5333(29.58)	
35–49	602(9.86)	634(10.78)	1011(16.74)	2247(12.46)	< 0.001
**Preceding birth interval**					
< 24months	290(4.74)	193(3.28)	244(4.03)	726(4.03)	
>=24months	1917(31.36)	2406(40.89)	2689(44.54)	7012(38.88)	< 0.001
**Birth order**					
1st	614(10.04)	789(13.41)	762(12.62)	2165(12.01)	
2nd-3rd	739(12.09)	1043(17.72)	1292(21.4)	3073(17.04)	
4th&above	854(13.98)	767(13.03)	879(14.56)	2500(13.86)	0.0039
**Child wantedness**					
Wanted then	1364(22.33)	1716(29.16)	1956(32.41)	5036(27.93)	
Wanted later	542(8.87)	603(10.26)	683(11.31)	1828(10.14)	
Wanted no more	301(4.92)	279(4.74)	293(4.86)	873(4.84)	0.548
**Sex of newborn**					
Male	1106(18.1)	1306(22.19)	1471(24.36)	3883(21.53)	
Female	1100(18)	1293(21.98)	1462(24.21)	3855(21.38)	0.062
**Iron supplementation**					
No	466(7.63)	438(7.45)	400(6.62)	1304(7.23)	
Yes	1741(28.48)	2160(36.72)	2533(41.96)	6434(35.68)	0.347
**Two tetanus injections**					
No	1290(21.23)	1605(27.35)	1762(29.27)	4657(25.92)	
Yes	903(14.86)	988(16.84)	1161(19.28)	3052(16.99)	0.661
**Low birthweight**					
No	2121(34.7)	2510(42.66)	2795(46.3)	7426(41.18)	
Yes	86(1.41)	88(1.5)	138(2.28)	312(1.73)	< 0.001

### The interrelationship between adequate utilization of ANC and neonatal outcomes

**Fig. 2:**
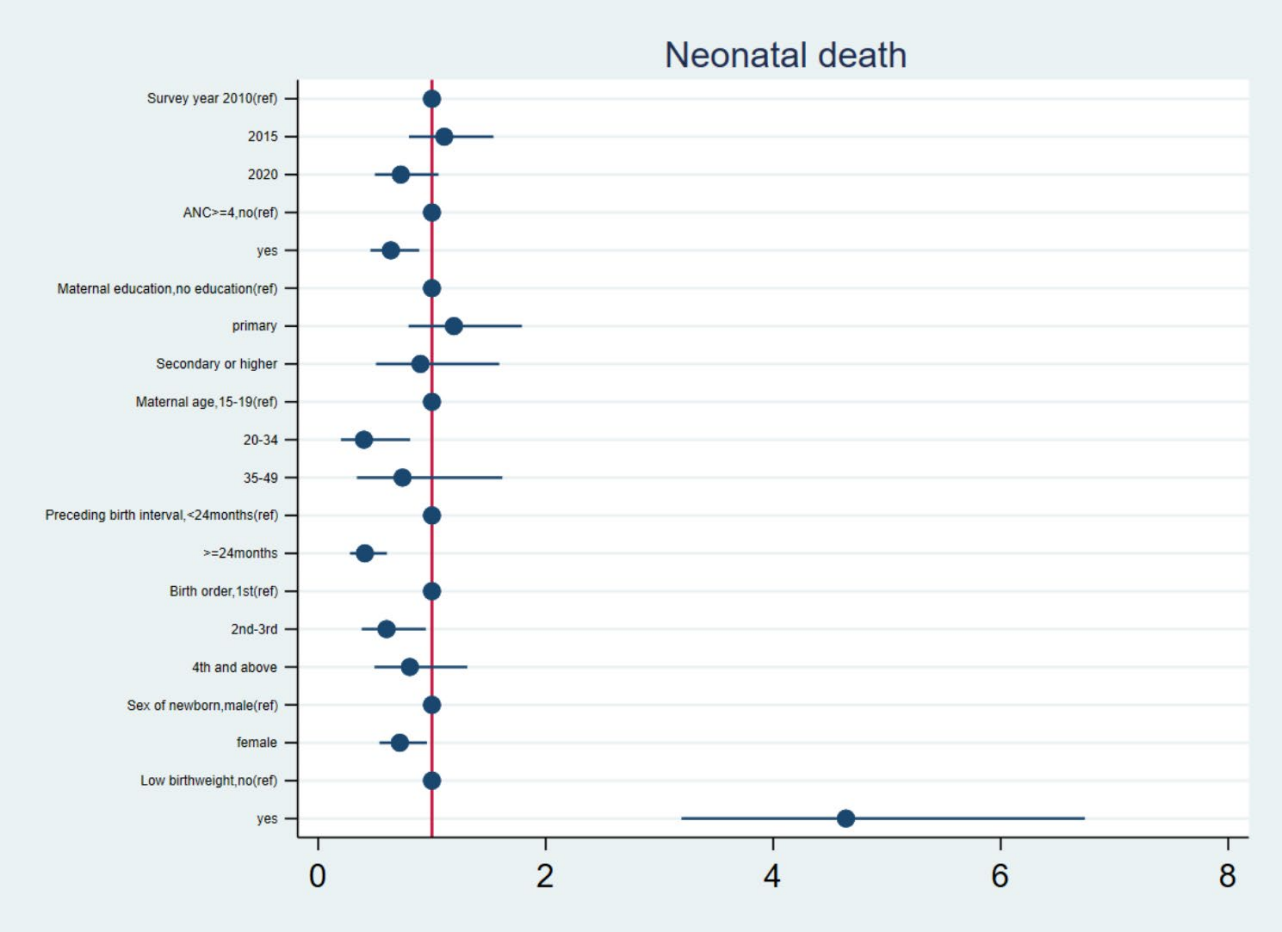
Adjusted odd ratios and 95%CI for multivariable analysis between adequate ANC and neonatal outcome

Figure 2 shows the details of the interrelationship between adequacy of ANC utilization and neonatal death. The predictors of neonatal death were having adequate ANC, young age of mother, the preceding birth interval, the birth order and low birthweight. Young mothers had 60% decreased odds of neonatal death (aOR:0.40; 95%CI:0.20,0.81) compared to the adolescent mothers. Having adequate ANC visits decreased the odds of neonatal death by 36%(aOR:0.64;95% CI:0.46,0.89) compared to inadequate use of ANC.A preceding birth interval greater or equal to 24months reduced the odds of neonatal death by 59%(aOR:0.41; 95%CI:0.28,0.60) compared to a preceding birth interval of less than 24months. A second or third birth order reduced the odds of neonatal death by 40%(OR:0.60;95%CI:0.38,0.95) compared to the 1st birth order. Sex of newborn being female reduced the odds of neonatal death by 28%(aOR:0.72; 95%CI:0.54,0.96) compared to male newborns. Low birthweight increased the odds of neonatal death by almost five times (aOR:4.64; 95%CI:3.19,6.74) compared to normal birthweight newborns.

## Discussion

The current study investigated the relationship between the adequate utilization of ANC and neonatal death in Rwanda. We found that the adequate utilization of ANC reduced by 41% the odds of neonatal death compared to the inadequate utilization of ANC.

The predictors of neonatal death were the adequate utilization of ANC, the age of the mother, the preceding birth interval, the birth order of the newborn, the sex of the newborn and low birthweight. When compared to inadequate ANC visits, adequate ANC visits had a lower risk of neonatal death. A recent hospital-based study in Rwanda discovered that women who received four or more ANC visits had a decreased incidence of LBW [30]. Several research have shown similar conclusions [20, 31–33]. A case-control study conducted in Brazil by Kassar S.B et al. in 2013 [20] and a case control study conducted in Ethiopia by Desta S.A et al. in 2020 [32] ound that four or more ANC visits were associated with a decreased risk in LBW. A cross-sectional study conducted in Bangladesh by Selina K et al. in 2008 [31] and a cross-sectional study in northern Uganda by Kananura et al. in 2017 [33] revealed that the increase in ANC visits were associated with normal birth weight. The World Health Organization (WHO) recommends at least four ANC checkups throughout pregnancy since this is a time when babies are vulnerable to issues such as preterm birth, restricted fetal growth, and congenital infections, all of which increase the likelihood of neonatal death [34]. In addition, attending ANC has been suggested as a possible avenue for mothers and their families to receive information and advice on obstetric care as well as the identification and management of infections such as Malaria, HIV/AIDS, syphilis, and other sexually transmitted diseases that affect the fetus [34]. This emphasizes the necessity of implementing population-based interventions that promote early ANC attendance [35].

In comparison to mothers aged 15–19, mothers aged 20–34 have a lower risk of newborn death. Studies in Ethiopia [36], Bangladesh, and Nepal have all reported similar results [37, 38]. Preterm birth or small-for-gestational-age are plausible mediating factors, according to a study conducted in Nepal [38], although neither of these variables were examined in our study. It’s also likely that a young mother’s perceived lack of childcare expertise is detrimental to their children’s health and survival [39]. The only way to effectively provide ANC for vulnerable populations is to integrate it with community-based public health programmes that target socioeconomic and lifestyle issues, such as youth care and social services [40].

In our research, preceding birth intervals of two years or more were found to less likely result in neonatal death than birth intervals of less than two years. This conclusion is in line with what has been discovered in Ethiopia and other developing Asian countries [36, 41–43]. The shorter birth interval impact could be linked to maternal depletion syndrome and resource sharing among siblings, as well as lower levels of care and attention for high-ranking infants [44, 45].

Our study revealed that LBW newborns were more likely to die during the neonatal period. Several studies have found that LBW babies are more likely to die [20, 46, 47]. According to these research, LBW babies are at a higher risk of a variety of health issues, which increases their chances of dying if proper care is not provided. Furthermore, the inadequate capacity of health services in LMICS to prevent, screen, and manage the issues associated with LBW babies makes it difficult for newborns to survive. As a result, interventions aiming at increasing health workers’ capacity to detect and care for LBW babies at health facilities are critical. LBW a leading cause of neonatal death could be due to small for gestational age, a variable that we were not able to capture in this study. perinatal and later adult health are significantly impacted by ANC. Maternal health and pregnancy outcomes could be significantly enhanced by health education aimed at adolescent girls, women, and their partners regarding ANC.

### Strengths and limitations

The study’s use of a nationally representative population-based, combined dataset is a notable strength. We were able to generate a large sample size by merging the three surveys, which allowed us to assess the impact of various factors on neonatal outcomes with acceptable precision. Another feature of the study is that the DHS 5-year birth history data provide good to excellent information for estimating child mortality [48, 49]; this has been proven to lessen recall bias in the interview mothers’ reporting of birth and death dates [50, 51]. Decision-makers can benefit from the evidence-based information this study offered for implementing public health policies pertaining to ANC evaluations and improvement. Our study, on the other hand, contains a number of limitations. In the DHS, only surviving mothers were interviewed, and there could be an association between maternal death and newborn mortality, resulting in an underestimating of neonatal deaths. Data on key major determinants of maternal health-care consumption, such as health insurance, was only gathered for the most recent delivery, which limited our ability to assess the impact of such variables. Variables such as facility readiness, interpersonal relationships between clinicians and women, transportation, and other cultural norms and beliefs that could have influenced the uptake of antenatal care were not included in this study. Due to the cross-sectional nature of the data, we were only able to investigate relationships rather than causality.

## Conclusion

Our findings demonstrate that the uptake of ANC has increased, which has improved newborn survival. Among the newborn survival interventions, addressing short birth intervals and the effective management of LBW cases should be explored. Addressing the coverage but also the quality of the content in ANC but also the skilling of the healthcare workers in dealing with risky obstetric conditions and equipping the health facilities with the essential tools for delivery care can potentially contribute to Rwanda’s achievement of the SDG goal 3.2 by 2030. Future research would carry out a longitudinal study examining the effect of several mediator variables from the ANC utilization to neonatal survival.

### Electronic supplementary material

Below is the link to the electronic supplementary material.

**Supplementary Material 1:** Crude and adjusted odds ratios for the relationship between adequate ANC and neonatal outcome.

## Data Availability

The datasets used during the current study are available from the DHS program website http://dhsprogram.com/data/available-datasets.cfm. but registration and application is required before access to data is granted.

## Abbreviations

ANC: Antenatal Care
RDHS: Rwanda Demographic and Health Survey
WHO: World Health Organization
LBW: Low birth weight
SDGs: Sustainable Development Goals
