# Supplementary material for: Association between adequacy of antenatal care and neonatal outcomes in Rwanda: a cross-sectional study design using the Rwanda demographic and health surveys
Source: BMC Health Serv Res. 2023 Dec 8;23:1379. doi: 10.1186/s12913-023-10345-6 (PMC10704762; doi:10.1186/s12913-023-10345-6)
Supplement: Supplementary file 1 — Supplementary Material 1: Crude and adjusted odds ratios for the relationship between adequate ANC and neonatal outcome. [file 12913_2023_10345_MOESM1_ESM.docx]

**Table S1: Crude and adjusted odds ratios for the relationship between adequate ANC and neonatal outcome**

|  | **Neonatal death** | **Neonatal death** |
| --- | --- | --- |
| **Variables** | **OR,95%CI** | **aOR,95%CI** |
| **Survey year** |  |  |
| 2010 | ref | ref |
| 2015 | 1.04(0.75,1.45) | 1.11(0.80,1.54) |
| 2020 | 0.71(0.49,1.03) | 0.73(0.50,1.06) |
| **Adequate ANC** |  |  |
| **no** | ref | ref |
| **yes** | 0.57(0.41,0.79) | 0.64(0.46,0.89) |
| **Type of residence** |  |  |
| Urban | ref | - |
| Rural | 1.09(0.74,1.59) | - |
| **Water sources** |  |  |
| improved | ref | - |
| unimproved | 0.82(0.58,1.15) | - |
| **Maternal education** |  |  |
| No education | ref | ref |
| primary | 1.07(0.72,1.60) | 1.19(0.79,1.79) |
| secondary&higher | 0.70(0.41,1.20) | 0.90(0.51,1.59) |
| **Married/partnered** |  |  |
| no | ref | - |
| yes | 0.79(0.55,1.14) | - |
| **Access to media** |  |  |
| not at all | ref | - |
| less than once a week | 1.18(0.72,1.94) | - |
| at least once a week | 1.25(0.80,1.93) | - |
| **Wealth index** |  |  |
| poor | ref | - |
| middle | 0.99(0.68,1.44) | - |
| rich | 0.97(0.71,1.32) | - |
| **Cooking fuel** |  |  |
| solid fuel | ref | - |
| non-solid fuel | 1.19(0.83,1.70) | - |
| **Maternal age** |  |  |
| 15-19 | ref | ref |
| 20-34 | 0.32(0.17,0.63) | 0.40(0.20,0.81) |
| 35-49 | 0.57(0.29,1.11) | 0.74(0.34,1.62) |
| **Preceding birth interval** |  |  |
| <24months | ref | ref |
| >=24months | 0.49(0.34,0.71) | 0.41(0.28,0.60) |
| **Birth order** |  |  |
| 1st | ref | ref |
| 2nd-3rd | 0.67(0.45,1.00) | 0.60(0.38,0.95) |
| 4th&above | 1.21(0.85,1.73) | 0.81(0.50,1.31) |
| **Child wantedness** |  |  |
| wanted then | ref | - |
| wanted later | 0.86(0.61,1.21) | - |
| wanted no more | 1.10(0.73,1.66) | - |
| **Sex of newborn** |  |  |
| male | ref | ref |
| female | 0.76(0.57,1.01) | 0.72(0.54,0.96) |
| **Iron supplementation** |  |  |
| no | ref | - |
| yes | 0.85(0.62,1.19) | - |
| **Two tetanus injections** |  |  |
| no | ref | - |
| yes | 0.93(0.69,1.27) | - |
| **Low birthweight** |  |  |
| no | ref | ref |
| yes | 4.5(3.1,6.54) | 4.64(3.19,6.74) |

-denotes not considered
